# Sustainable education and youth confidence as pillars of future civil society

**DOI:** 10.1038/s41598-023-28143-9

**Published:** 2023-01-18

**Authors:** Alberto Biancardi, Annarita Colasante, Idiano D’Adamo

**Affiliations:** 1 Department Studies, Monitoring and International Relations, Gestore dei Servizi Energetici GSE S.P.A., Rome, Italy; 2 Department of Law and Economics, UnitelmaSapienza University of Rome, Rome, Italy; 3 Department of Computer, Control and Management Engineering, Sapienza University of Rome, Rome, Italy

**Keywords:** Environmental social sciences, Sustainability

## Abstract

While sustainability is at the centre of many government agendas, there is a great risk of entrusting strategic decisions to those lacking in sustainability expertise. It is therefore necessary to ensure that universities are the green engines of sustainable communities. The present study administered a questionnaire to students enrolled in a Management Engineering programme at an Italian university, to collect their perceptions of and opinions on sustainability and energy issues. Students completed the questionnaire twice: once prior to beginning and once at the end of term. The results showed that students held more sustainable attitudes at the end of term, and perceived sustainable education and youth confidence as the building blocks of future society. They also observed that decarbonisation of the Italian energy system and national energy independence would require the significant development of renewable systems and interventions to promote energy efficiency. In addition, they recognised subsidies for green production, energy communities, differentiated waste collection and professional skills training as crucial. The sustainable university should support younger generations by encouraging student engagement in real-world projects and the development of long-term, structured teacher–student relationships.

## Introduction

Amidst ongoing unsettling events, including a global pandemic and a war in Ukraine, the challenge of sustainability remains central^1,2^. In addition, there are many problems related to health, human rights and the environment, including malnutrition and food insecurity^3^, as well as water shortage^4,5^. The Sustainable Development Goals (SDGs) offer a pathway to reconciling economic growth with environmental concerns, through a highly interconnected framework of analysis^6^. All countries must develop policies and trackable objectives to support the achievement of these goals^7^.

The dilemma of intergenerational sustainability refers to the challenge of determining whether one will sacrifice in the present for the sake of future generations^8^. On World Youth Day in 2000, Pope John Paul II defined sustainability as the pursuit of the unselfish^7^, with the following call to action: ‘You will defend life at every moment of its earthly development, you will strive with all your energy to make this earth more and more habitable for all’. Around the world, universities and research centres have taken up this call; and while much progress has been made, more must be done to support young sustainability scholars, whose work is often team-based and outreach-oriented^9^.

While sustainability is not a new topic in the literature^10,11^, a framework is still needed to comprehensively cover all 17 SDGs and their multiple areas of analysis^12^. Some authors have proposed a tetrahedron framework with students positioned at the centre, and student competence, teaching methodology, professors and alliances placed at the vertices^13^. Generally, institutional initiatives and campus operations are the two main channels through which higher education institutions (HEIs) promote sustainability^14^.

Previous research has attempted to develop methodologies to assess sustainability learning practices in HEIs^15^. In particular, scholars have emphasised the ‘living lab’ model, which involves both top-down and bottom-up strategies^16^. This implies that the implementation of SDGs at the university level should be driven by institutional, thematic, structural and personal/individual forces^17^. Additionally, some studies have underlined that the online presence of universities, the internationalisation of universities, and financial resources for research and infrastructure from regional governments are relevant internal/external factors for achieving the SDGs^18^. Students’ collaborative problem-solving competency may also play an important role^19^.

Universities may promote transformative innovation^20^ through their curricula, as well as by engaging in joint initiatives with the local community^21^. Indeed, sustainable entrepreneurship support programmes linked with universities have the potential to spill over into regional development^22^. Furthermore, educational and extracurricular programmes are helpful for promoting students’ green skills^23^. Several studies have underlined the need to strengthen students’ knowledge and proactivity regarding sustainable development^24^, and a related need to develop appropriate curricula and government initiatives to address the green transition^25^. These findings bear particular weight for university students in scientific programmes^26^ such as engineering^27^, as such students may underestimate their role in addressing social inequalities. Thus, sustainability should be discussed within these programmes in order to introduce students to the challenges they are likely to face in their professional work^28^.

In line with this, the present study measured the impact of a sustainability course on student perceptions at an Italian university. Data were collected by means of a questionnaire that was administered twice: once prior to and once at the end of the course. The questionnaire aimed at collecting student opinions on and perceptions of sustainability issues relevant to the material discussed in the course.

## Methods

The present study adopted a behavioural methodology, in line with the classification proposed by Sovacool et al.^29^. More specifically, it took a multidisciplinary approach, fusing ideas from numerous disciplines (e.g., psychology, engineering, economics). Questionnaires are commonly implemented to assess consumer^30,31^ and producer^32^ attitudes and behaviours in many fields (see, e.g., Balest & Stawinoga^33^; Svenningsson et al.^34^). Similar to Menon and Suresh^35^, the present study deployed this methodology to collect information from a sample of students.

The investigation proceeded across four phases: (i) a first draft of the questionnaire was written, based on the relevant literature; (ii) the questionnaire was validated; (iii) the questionnaire was administered to students prior to and at the end of the course; and (iv) a panel discussed the main results. The first two phases occurred *ex ante* (i.e., pre–data collection). The initial questionnaire was reviewed by a small panel of experts/academics in the field of sustainable management. This panel was selected from the Scopus database, which verified panellists’ high scientific impact and experience (of at least 10 years). The panel validated the questionnaire items, in terms of their relevance to the topic and clarity, as well as the entire questionnaire, in terms of its overall length. Based on the panel feedback, some items were eliminated and minor changes were made.

Once the questionnaire was finalised, it was administered to a sample of 66 students enrolled in a master’s degree programme (mainly the Management Engineering) at the University of Rome La Sapienza. Specifically, students were taking an optional course with a strong focus on sustainability, entitled ‘Economics and Management of Energy Sources and Services’. Students were asked to complete the questionnaire twice: (1) prior to beginning and (2) after completing the full course. The aim was to observe whether attending the course significantly affected students’ sustainability attitudes and behaviours. Though it was not possible to control for previous knowledge, the questionnaire aimed at assessing the ‘treatment effect’ stimulated by the information provided during the course. Therefore, it was assumed that any background knowledge possessed by students would play a marginal role in determining the main findings, consistent with the transformative learning approach^36^. Following the final administration of the questionnaire, some of the main results were presented to the students, stimulating discussion and debate that produced further insight into students’ reasoning.

Students’ average age was 23.7 years, and approximately 64% were male; 39% were student-workers and 82% lived with their families of origin. With respect to their geographical place of origin, 85% came from the central macro-region of Italy (see questions 1–6 in Supplementary Appendix A). Data were collected by means of a questionnaire with approximately 40 items (reported in Supplementary Appendix B and discussed in the following section). The questionnaire was sent to students via email, and students were given 5 days to complete it. The data collection proceeded in two phases: in the pre-course phase, students answered the questionnaire based on their prior knowledge; in the post-course phase, students answered the same questionnaire with the information they had gained in the course. Following the post-course phase (i.e., in the final class session), responses were discussed with the students to collect further qualitative data. Questionnaires were administered in February and May 2022.

### Ethics statement

Given that the research is a non-experimental voluntary survey, no ethical approval is necessary. Furthermore, the self-administered survey that is non-experimental in nature was conducted under complete anonymity for the participants. No personal or sensitive information that can be used to identify the respondents were collected. Besides, the consent of the respondents to partake in the online survey were seek before the survey was executed by including an electronic informed consent in the online survey form.

## Results

The present study combined a conventional methodological approach with an innovative approach to application. While the concept of sustainability evokes opportunities for younger generations, the needs and opinions of youths are not always heard, and issues around sustainability have only recently gained space within educational curricula. Thus, the present study administered a questionnaire to explore how and whether students’ responses changed after taking a course on sustainability. All questionnaire items pertained to topics that were covered in the educational course.

After the post-course phase, the results were presented to and discussed with the students. During this discussion, the students expressed a strong interest in sustainability, whilst emphasising its complexity. They decried rhetoric, common phrases and projects in which sustainability is discussed without practical application. They also understood that, while the sustainability challenge is not simple, it is crucial for the future, particularly in light of European policies. They were ready to support change if they were given the tools and could develop the skills to meet the challenge. While the remainder of this section analyses students’ questionnaire responses in detail, in general, students’ attitudes at the end of the course were more aligned with sustainability, relative to their attitudes at the beginning of the course. Supplementary Appendix B presents all of the questionnaire items, together with the student responses.

### General data

The first block of items concerned general sustainability (e.g., the definition of sustainability) and its behavioural aspects (e.g., individual sustainability behaviours). In general, many individuals are unclear about the meaning of sustainability, tending to link it to only the environment^37–39^. Therefore, the course curriculum sought to clarify that sustainability has three dimensions: social, environmental and economic. While the vast majority of the students were clear at the beginning of the course that sustainability encompasses these three spheres, approximately 9% were convinced that it only involved environmental aspects; however, these students changed their opinion after taking the course (see question 9 in Supplementary Appendix A).

### Sustainability behaviours

**Figure 1:**
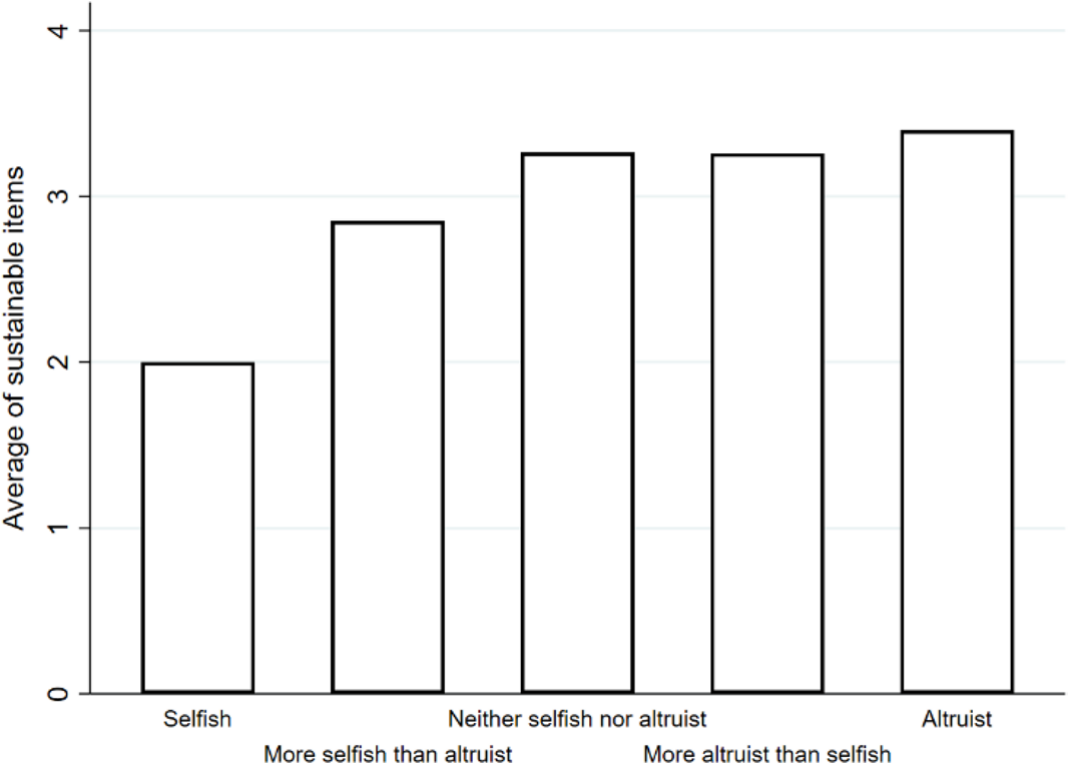
Mean values for each degree of altruism, in relation to the index of sustainable behaviours.

The literature shows that pro-environmental attitudes relate to certain character aspects, including altruism^40,41^. Therefore, students were asked to assess their degree of altruism. Approximately 75% described themselves as more altruistic than selfish (see question 7 in supplementary Appendix A). Additionally, six questionnaire items were designed to measure students’ sustainable behaviours (i.e., volunteering, playing sports, engaging with nature, using sustainable products, recycling, and taking public transportation. See questions 10–16 in supplementary Appendix A). The decision to include these items was based on previous research showing associations between: (i) playing sports and volunteering and greater sustainable behaviour^42,43^, and (ii) engagement with nature and improved stakeholder engagement^44^; as well as the use of recycling, using sustainable products and taking public transportation as proxies of pro-environmental attitudes^45^. An index was created to average student scores on these six items and assess any correlation with character aspects (i.e., altruism). As seen in Fig. 1, a higher degree of altruism corresponded with greater sustainable behaviours.

**Figure 2:**
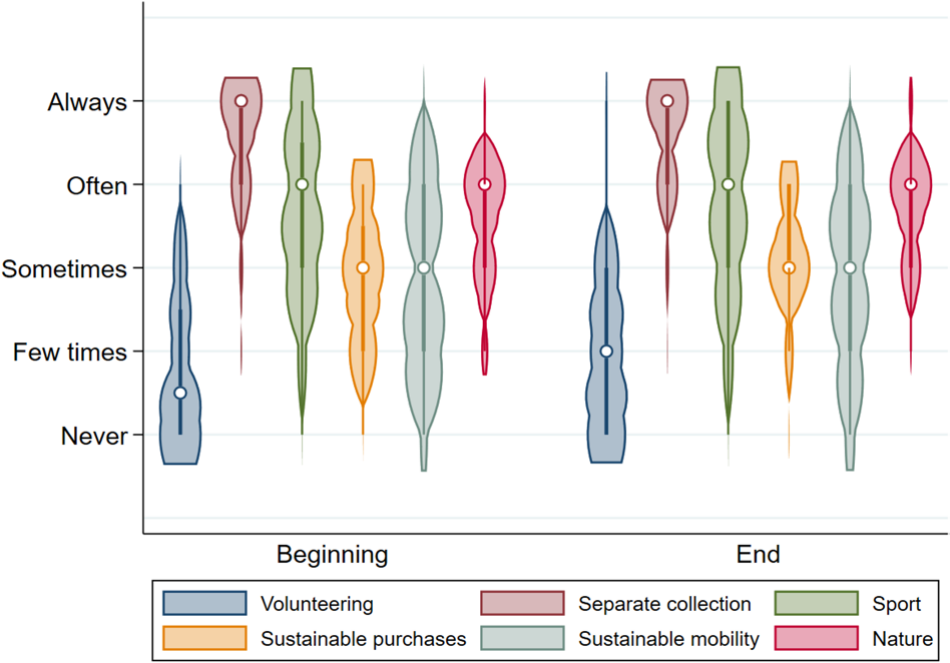
Distribution of responses related to sustainability behaviours, divided between the beginning and the end of term. White dots represent median values. Values at the beginning of term were (mean values in parentheses): 1.5 (1.8) for volunteering, 5 (4.3) for recycling, 4 (3.7) for playing sports, 3 (2.9) for using sustainable products, 3 (2.8) for taking public transportation, and 4 (3.5) for engaging with nature. Values at the end of term were (mean values in parentheses): 2 (1.9) for volunteering, 5 (4.5) for recycling, 4 (3.8) for playing sports, 3 (3.1) for using sustainable products, 3 (2.9) for taking public transportation, and 4 (3.7) for engaging with nature.

In more detail, the analysis sought to uncover whether and how the distribution of responses related to individual items changed after the course (all responses were measured using a Likert scale ranging from 1 (*never*) to 5 (*always*)) (see Fig. 2).

The only significant change emerged in the use of sustainable products, for which average scores increased from 2.9 to 3.1 over the course period. The main reason offered for the infrequent use of sustainable products was their prohibitive cost. In fact, students perceived these items as ‘luxury goods’ and, generally, such products were only used by students with medium to high incomes. The correlation between income and the use of sustainable products has already been noted in the literature^46,47^, and there is a risk that, without effective interventions, sustainable products will only be purchased by those in higher income brackets.

Concerning the other sustainable behaviours, the course did not seem to have a significant impact, as the mean values remained virtually unchanged. However, it is important to note that most students claimed to frequently sort their waste (4.5). This is an interesting finding, since it was obtained in Rome, where waste management performance is unsatisfactory. In particular, there is a need in Rome for both on-site facilities for waste disposal and a significant reduction in the amount of waste delivered outside the region^48^. Finally, the intermediate rating (2.9) recorded for the use of public transportation can be read in several ways: (i) the pandemic may have led many students to reduce their use of very crowded modes of transportation, and (ii) students may have been discouraged from using public transportation due to the poor time reliability.

Because sustainability is relevant to present and future generations, students were asked whether they were, in general, more anchored in the present or projected into the future. The majority (64%) of students tended to look to the future (see question 8 in supplementary Appendix A). Sustainability behaviours do not preclude living in the present, but merely call for a frugal attitude, so that resources can be maintained for future generations.

### Economics of sustainable energy

The second block of questionnaire items concerned more specific energy issues discussed in the course. These items assessed students’ willingness to pay (WTP) for green items and their particular behaviours as both producers and consumers (see questions 17–20 in supplementary Appendix A). A scenario was given in which a kWh of energy obtained from renewable sources was sold at 19.1 cent€/kWh, with a green premium of 2.4 cent€/kWh. In a second scenario, a kWh of energy from renewable sources was sold at 20.2 cent€/kWh, with a green premium of 6.6 cent€/kWh. These scenarios were based on the price conditions that existed prior to the war between Russia and Ukraine.

**Figure 3:**
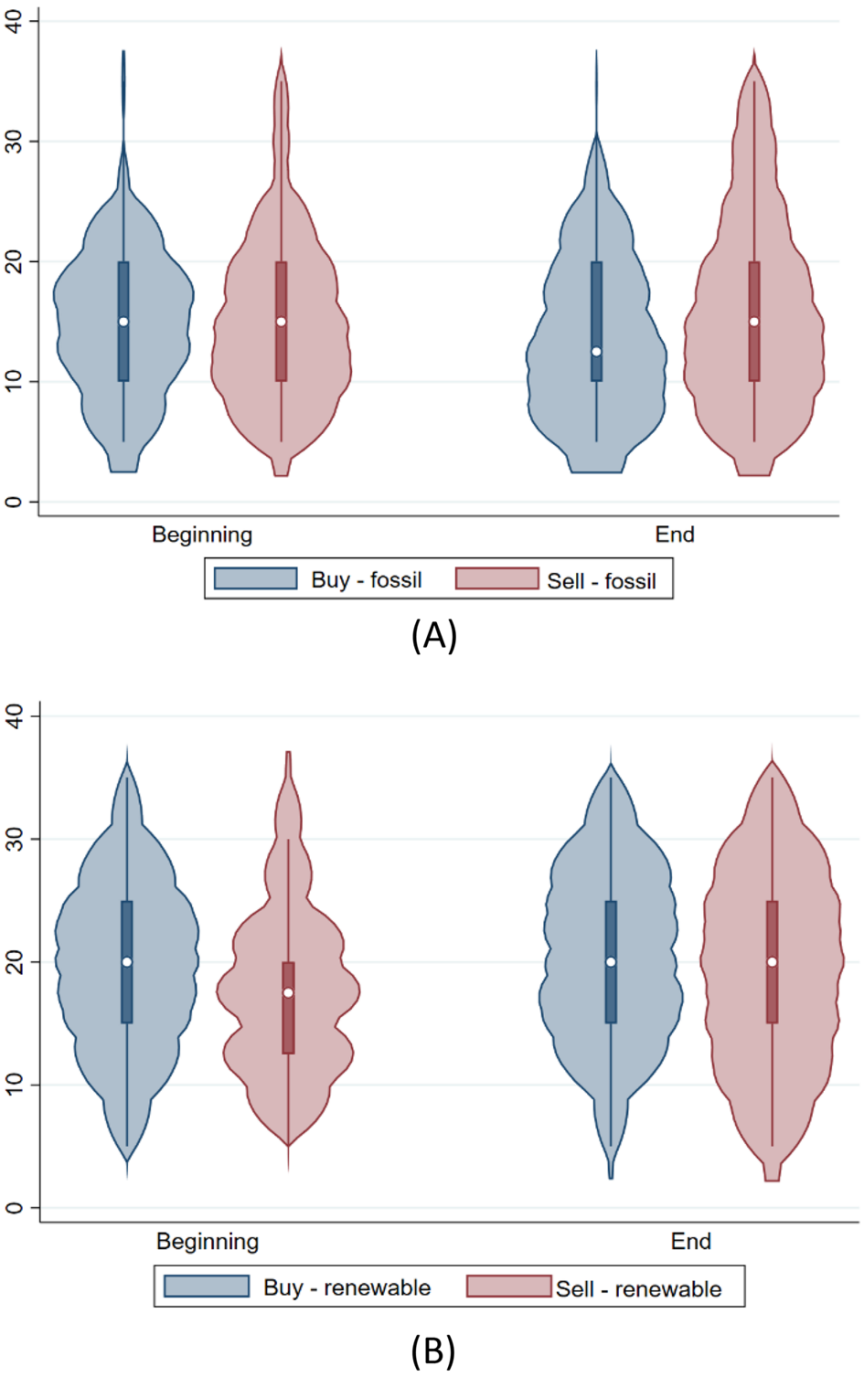
WTP values for the purchase and sale of energy produced from fossil sources (**A**) and renewable sources (**B**).

Figure 3 shows how students changed their evaluations of these scenarios between the beginning and the end of the course. In particular, three important findings emerged. First, at the beginning of the course, students assigned, on average, the same price for buying and selling fossil energy (panel A; for fossil energy, the initial and final average values were 15.3 and 13.6 cent€/kWh, respectively, for purchasing; and 16.1 and 16.7 cent€/kWh, respectively, for selling). This signals misinformation related to the price difference between purchased and sold energy. At the end of the course, students evaluated this type of energy as ‘inferior’, recording a lower WTP than both the initially stated WTP and the WTP for renewables (panel B; for renewable energy, the initial and final average values were 19.6 and 20.2 cent€/kWh, respectively, for purchasing; and 18.0 and 19.1 cent€/kWh, respectively, for selling).

Second, there was no significant variation between students’ WTP for renewable energy between the beginning and the end of the course. This implies that students were able to assign the correct value to this type of energy from the beginning. Third, the WTP value of 6.6 cent€/kWh that emerged in the present study is slightly lower than the values of 8 cent€/kWh and 10 cent€/kWh that were previously recorded in Spain and Italy, respectively^49^. However the study conducted in Spain and Italy considered a reference sample characterised by older individuals. The fact that students mainly lived with their families and did not pay their energy bills themselves likely influenced the present result.

### Subsidies for energy

**Figure 4:**
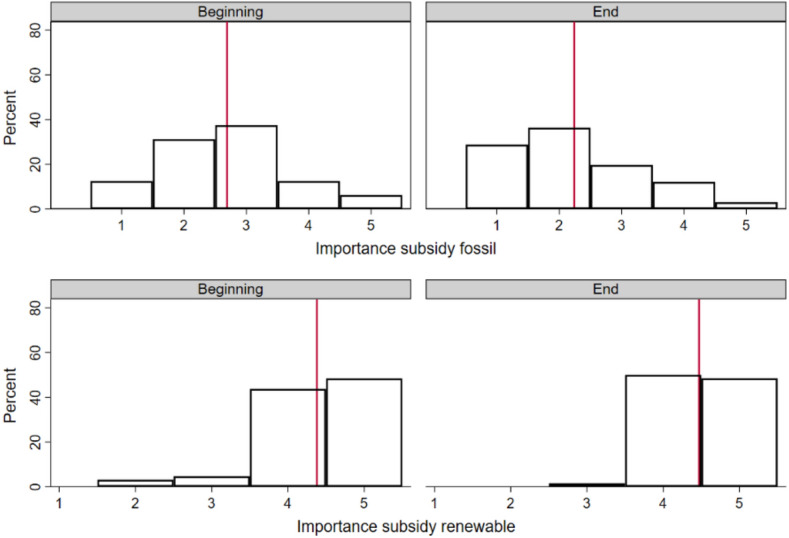
Distribution of responses on the importance of subsidies for the production of energy from fossil or renewable sources. Red lines represent the mean values of 2.7 (beginning) and 2.2 (end) for energy from fossil sources and 4.4 (beginning) and 4.5 (end) for energy from renewable sources.

Subsidies are a controversial topic. On the one hand, they are deemed necessary for the green transition, by reducing production costs for sustainable items to a point that they are competitive with those of fossil-based products (without considering externalities). On the other hand, their use must be metred to prevent any increase in public debt or the price paid by the final consumer. Students were asked to express their opinion on a 5-point scale about the importance of subsidies for both renewable and fossil-based energy (see questions 21–24 in supplementary Appendix A). In addition, they were asked to report the extent to which they agreed (on a 5-point scale) that they should produce energy themselves, in the absence of subsidies (see question 40 in supplementary Appendix A). Students indicated that they were generally ‘undecided’ on this latter point, though their indecision fell at the maximum value of the range (3.5). Regarding their evaluation of subsidies, they assigned a high value to subsidies for energy from renewable sources (4.5). For energy from fossil sources, their approval for subsidies for self-consumption changed from ‘sometimes’ to ‘rarely’ (2.7 vs. 2.2); this variable showed the greatest change between the beginning and the end of the course (Fig. 4).

**Figure 5:**
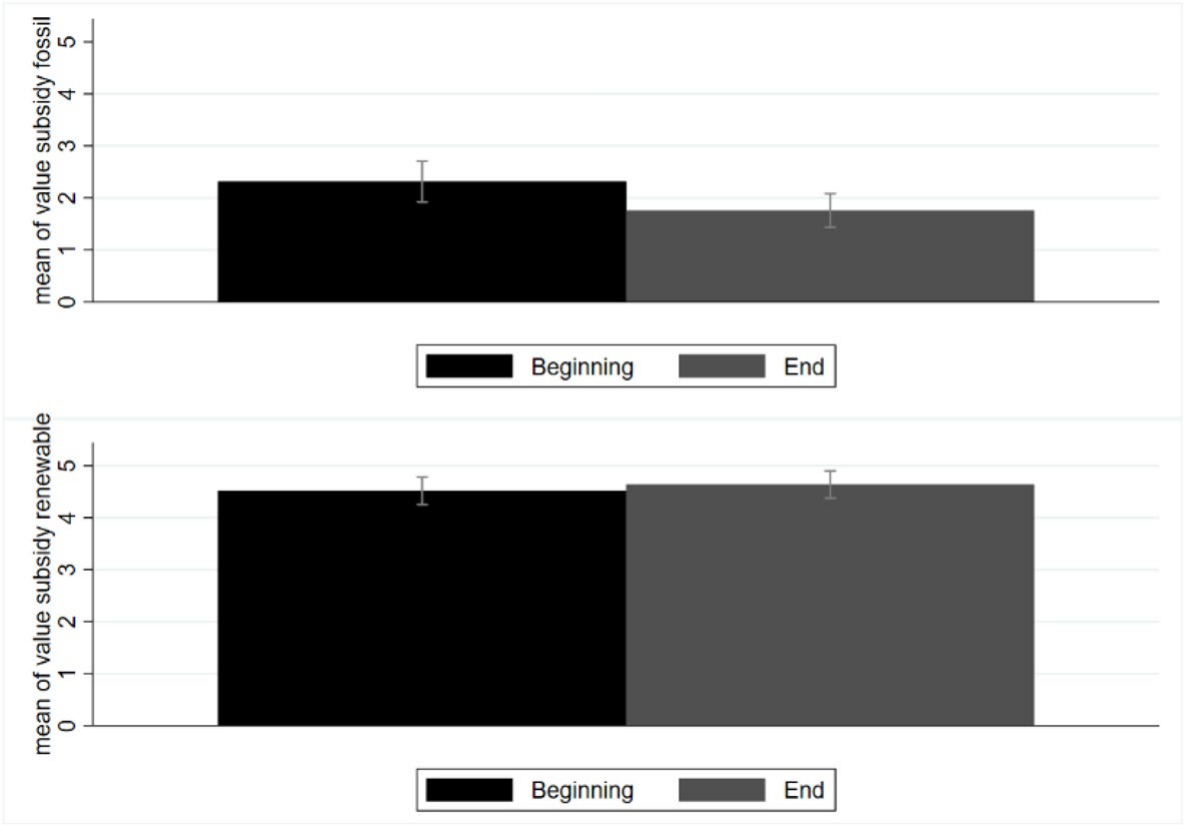
Average values assigned to subsidies for the self-consumption of energy from fossil and renewable sources. Black bars represent the average values at the beginning of the course and grey bars represent the average values at the end of the course. All values are expressed in cent€/kWh.

Students were also asked to estimate the appropriate subsidy levels (within a range of 0–6 cent€/kWh and see questions 25–26 in supplementary Appendix A). As can be seen in Fig. 5, any difference in opinion vanished when students assigned these numerical values.

Citizen involvement in the energy transition may require subsidies for energy that is produced and self-consumed. In the present study, this value was quantified as 4.6 cent€/kWh for energy from renewable sources and 1.8 cent€/kWh for energy from fossil sources (decreasing by 0.5 cent€/kWh, consistent with the previous figure). Interestingly, the subsidy for the self-consumption of renewable energy was valued as high from the beginning of the course, signalling students’ sensitivity to the issue of sustainable self-consumption. In a similar vein, the equivalent subsidy for energy from fossil sources was valued as low from the beginning, and it decreased at the end of the course. This implies that students became even more aware that energy from fossil sources should be disincentivised. However, the difference in value associated with the subsidies for the production and self-consumption of green energy versus fossil energy was 2.8 cent€/kWh. The literature reports higher values for subsidies for the self-consumption of renewable energy: 3 cent€/kWh for Spain and 4 cent€/kWh for Italy (of note, in this research, only subsidies for energy from renewable sources were claimed) ^49^.

The International Energy Agency reports that, globally, subsidies for fossil sources amount to approximately 400 billion USD, and action must be taken to reduce this sum. To support the green transition, the state of the art must be communicated more widely and subsidies must be established. Regarding taxes, the present study found that students favoured them for giving economic weight to externalities. In particular, students felt that penalties should be higher for businesses than for citizens (4.3 vs. 3.9 and see questions 29–30 in supplementary Appendix A). However, they considered both penalties significant, in alignment with the European Commission, which has placed green taxes at the centre of its agenda.

### Energy communities, sustainable certifications and sustainable competitive advantage

**Figure 6:**
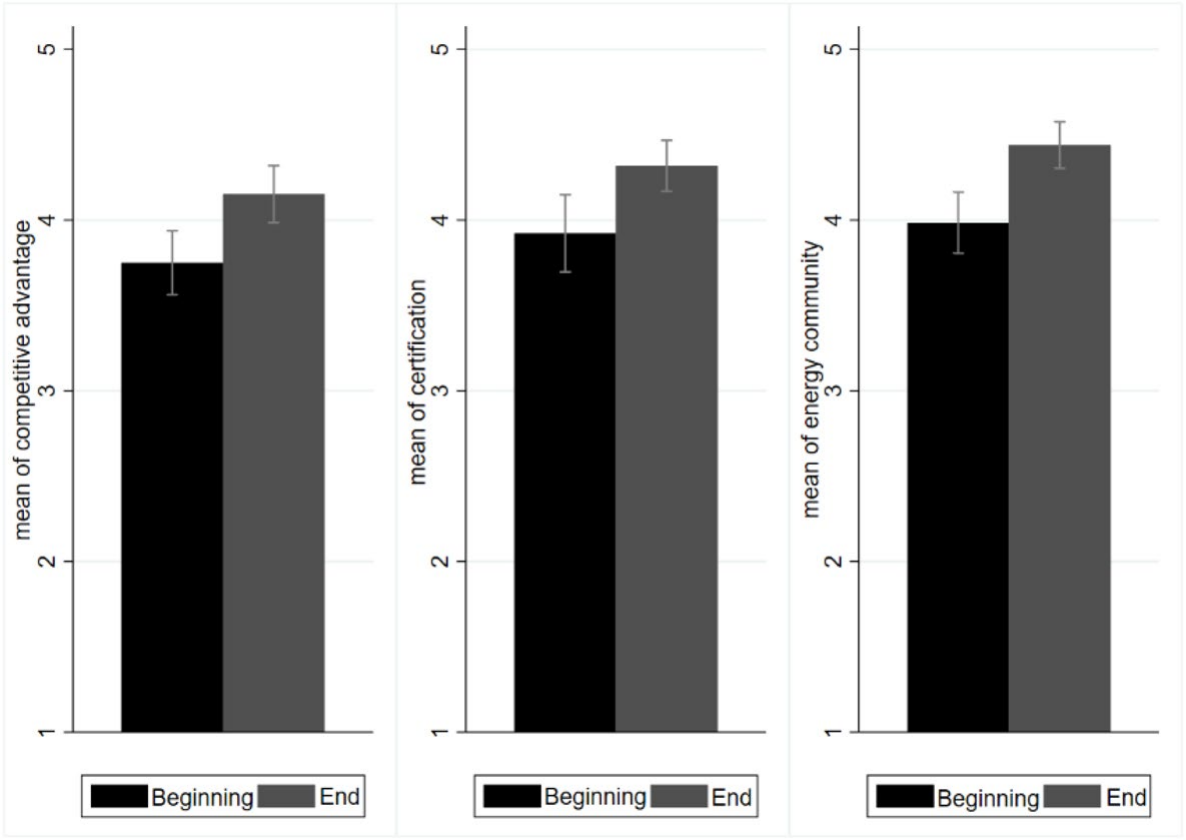
Mean values for the importance of energy communities, sustainable certifications and competitive advantage. Black bars represent values recorded at the beginning of the course and grey bars represent values recorded at the end of the course. Values range from 1 to 5, according to the Likert scale.

Students were also asked to express their opinion on the importance of energy communities, certifications and competitive advantages. These variables showed the same and a maximum increase between the beginning and the end of the course (+ 0.4) (see Fig. 6 and see questions 21, 22 and 28 in supplementary Appendix A).

Energy communities, while a fairly new concept, found enthusiastic support from students (i.e., registering an average value of 4 at the beginning and 4.4 at the end of the course). Stakeholder engagement, with shared models showing how the economic benefits are distributed, emerged as an enabling factor^50^. Regarding sustainable certifications, students had a clear understanding of these and, in fact, assigned them a very high value (i.e., 3.9 at the beginning and 4.3 at the end of the course). However, they noted that a limitation of such certifications is that their cost is typically passed on to the consumer. Certified products were not always linked to a higher WTP, especially for those in lower income brackets. Finally, students deemed the competitive advantage associated with the use of green sources very relevant (i.e., 3.8 at the beginning and 4.2 at the end of the course). The beginning of the war in Ukraine coincided with the course, and classroom reflections emphasised that individuals and companies that had installed renewable energy systems were not only experiencing fewer spillover effects related to inflation, but also seeing increased savings on their energy bills.

### Energy-related policies

While a large proportion of engineering students (42%), who would be expected to support the use of technology, noted that current technologies should be sufficient to meet the sustainability challenge, an equal proportion of students saw an additional change in behaviour as necessary to decarbonize the Italian system (see question 42 in supplementary Appendix A). Accordingly, the students did not favour a policy based totally on electrification. Another highly debated topic was Italy’s strong energy dependence on foreign countries, and particularly Russia (noting that the funds that had previously been invested in foreign energy could have instead been invested in the development of green energy). For that reason, students were asked to assess (on a 5-point scale) the extent to which they considered the use of renewable sources important for mitigating geopolitical risk (see question 27 in supplementary Appendix A). Based on their responses, the students understood that some non-choices of the past had resulted in very large costs in the present. They recognised that short-sighted policies and ‘no’ committees—related to ‘not in my term of office’ (NIMTO) and ‘not in my back yard’ (NIMBY) attitudes—had resulted in significant costs of not doing. Thus, the idea emerged that geopolitical risks could be reduced through the use of renewables (3.9). However, in assessing whether the use of renewables could positively impact the environment (see question 32 in supplementary Appendix A), even though the students agreed that this was likely (4.4), they stressed that care must be taken to avoid an economic rebound effect, emphasizing that the use of green sources could not justify energy waste. At the start of the course, the students were generally ‘undecided’ about this aspect; while at the end of the course, the dominant attitude was ‘strongly disagree’ (2.3 vs. 2.7 and see question 35 in supplementary Appendix A). This value must still be reduced. Approximately 65% of the students considered energy efficiency interventions as relevant as the use of renewables in efforts to achieve climate neutrality (see question 41 in supplementary Appendix A). Furthermore, the students felt that green energy could contribute to changing consumption habits by exploiting economic benefits (3.9, see question 34 in supplementary Appendix A). In addition, approximately 80% of the students believed that society (in general) and value chain actors had the greatest impact on sustainable development (see question 43 in supplementary Appendix A). In contrast, approximately 18% placed the highest responsibility on consumers, while attributing minimal responsibility to the local community and no responsibility to workers.

### Green marketing

The most perplexing student response concerned greenwashing. In fact, students were asked whether greenwashing helps sustainable development (see question 39 in supplementary Appendix A). This question was originally included in the questionnaire to mislead students, since, upon first encounter, the term ‘greenwashing’ may suggest something positive to those who are unfamiliar with its true definition. The results showed that students’ attitudes shifted from 2.8 (i.e., ‘undecided’) at the beginning of the course to 2.4 (i.e., ‘strongly disagree’) at the end of the course. There are at least two potential interpretations for this. First, the finding may be interpreted as positive, as the value reduced over the course period and the final summary judgment also changed. However, the second interpretation is that the rating of 2.4 still represents an excessive value, in need of further reflection. Some students may not have wanted to offer their true opinion on an ‘uncomfortable’ topic, and instead provided an ‘uncomfortable’ answer under the conditions of anonymity, despite knowing the real meaning of the term greenwashing. This idea emerged during the discussion of the final results, in which no student offered a plausible explanation or justification of the greenwashing finding. Also noteworthy is the high rate of student absence (approximately 25%) on a weekly basis. However, this impact could not be measured, considering the anonymity with which the questionnaires were completed. In addition, regarding distance learning support, students considered the internet influential for sustainability (3.9, see question 38 in supplementary Appendix A). They also perceived digital development as necessary, elaborating that such development needed to be calibrated to actual need.

### Sustainable education

**Figure 7:**
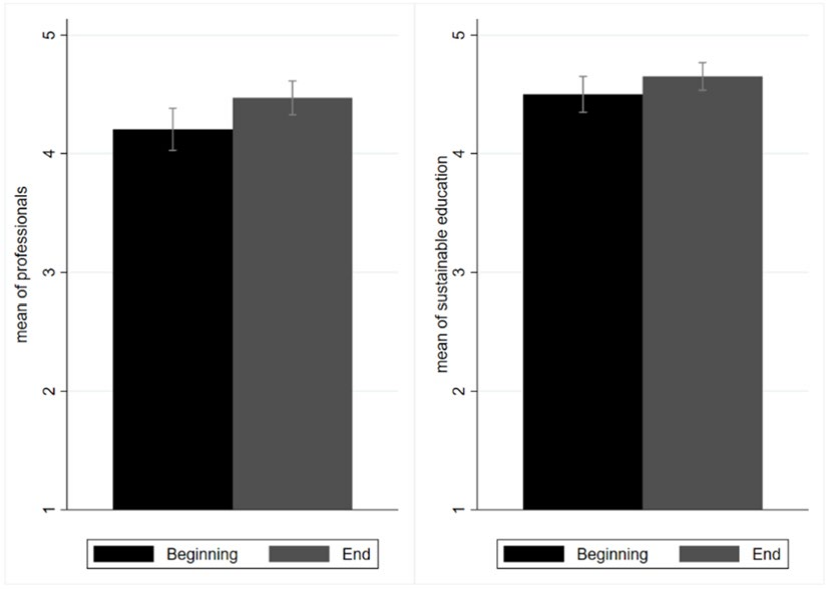
Mean values for sustainable education and the need for new professionals to support the green transition. Black bars represent values recorded at the beginning of the course and grey bars represent values recorded at the end of the course. Values range from 1 to 5, according to the Likert scale.

The importance of sustainable education received the most support (see question 31 in Supplementary Appendix A), and was the only variable that received a ‘fully agree’ rating (i.e., 4.5 at the beginning and 4.7 at the end of the course) (Fig. 7). This represents a core finding. The students also attributed much importance to the development of new professional figures (4.5, see question 33 in Supplementary Appendix A). In fact, this variable was rated as the second most relevant, alongside waste collection and subsidies for green energy. The students felt that sustainability courses might change production systems and make students more attractive on the job market. Finally, their perception of the relevance of the younger generation to sustainability was unexpected. Specifically, students were asked to report the extent to which they agreed that students are able to develop a sustainable plan (see questions 36–37 in Supplementary Appendix A), and they rated this factor as 3.6 with regards to university students and 2.9 with regards to high school students. This suggests that they lacked confidence in not only their current generation, but also the younger generation. Students elaborated that the course had underlined the complexity of sustainability and, as a result, they doubted that their peers were up to the challenge of realising it. In this regard, students emphasised the importance of sustainable education and the development of sustainability projects. However, they noted that the course they had just completed was an elective, and not mandatory. Also, the younger generation was perceived to have less knowledge about sustainability.

## Discussion

The present study, in accordance with the literature, emphasised the central role played by students in contributing to the sustainability challenge^13,51^. Specifically, the results showed that the sustainability course was essential for changing students’ sustainability attitudes and behaviours. The findings support the model of a sustainable community based on stakeholder engagement, which can fundamentally change even industrial systems^44^. However, sustainable communities require support from HEIs^26^, which in turn need to connect to the outside world^52^ and embrace change, on the basis of dynamic competencies. Some authors have suggested rethinking the role of universities within society^53^ by strengthening their connection with local realities in order to increase their resilience^54^, without neglecting the importance of their internalization^55^. In this vein, empathy and trust have been identified as critical for achieving a cooperative model^56^.

Works on sustainability often attend to the environmental component, first, before applying this to a fuller analysis. In fact, the quantitative approach is instrumental to a broader sustainability analysis^57^, by measuring the impact of a diversity of applications and the need for critical thinking^58^. This inevitably recalls the idea of creative abrasion as a model of innovation. However, new ideas and approaches are needed in order to increase sustainable skills^59,60^. The present work moves in a different direction, as most of the proposed values allow for evidence to be collected in relation to progress.

The choice to focus on energy was not secondary. By metaphor, a university course needs energy to be completed. While the literature shows the decisive role played by social trust in contributing to sustainability in Europe^61^, a recent study highlighted the need for new efforts in this context^62^. This latest work (similar to the present work) considered only a small reference sample. Nevertheless, tomorrow’s engineers will be required to not only problem solve^19^, but also to consider social dimensions^27^ in order to achieve models that meet the needs of both individuals and organizations. University education may play a significant role in developing these skills and competencies in the next generation^63^, by educating students on green issues^64^ and encouraging them to identify solutions to current problems.

## Conclusions

The sustainability challenge has not only led to a different approach to the creation of products and services, but it has also promoted the development of new social models. Previous research has explored the topic of sustainability within HEIs, where there are ongoing efforts to provide the right skills for future graduates. The present study focused on Management Engineering students at the University of Rome La Sapienza, who were enrolled in an Economics and Management of Energy Sources and Services course. The course curriculum introduced tools for addressing social inequalities, seizing economic opportunities and tackling environmental challenges within sustainable energy systems. In particular, management engineering students, educated on strategic, economic and management topics, have the potential to contribute significantly to the green transition, through their aptitude for problem-solving.

In the present study, students’ attitudes toward sustainability issues improved over the course. More specifically, several findings can be highlighted: (i) students were strongly interested in sustainability, but understood it to be highly complex; (ii) students did not link sustainability to a simple challenge; (iii) students associated sustainable products/services with higher prices, which they could not always afford; (iv) students’ engagement with nature was relevant to their sustainable attitudes, but could become less relevant over time, due to professional commitments; (v) students reported a WTP for green energy, whether produced and self-consumed or sold; (vi) students considered it appropriate to tax businesses and citizens for unsustainable behaviour; (vii) students considered subsidies important (particularly a bonus for the production and self-consumption of green energy); (viii) students considered energy communities central for the green transition; (ix) students did not necessarily report a higher WTP for products with a sustainable certification; (x) students recognised the need for domestic energy production; (xi) students acknowledged the need for changes in consumer habits; and (xii) students highlighted the need for the development of new professionals.

Sustainable education and the development of youth confidence in HEIs may encourage innovation in ecosystems and support the growth of local economies, through collaboration with businesses and public administrators. To support these ends, HEIs must not only update their educational curricula, but also strengthen their connection with the outside world. The complexity of the sustainability challenge can only be addressed through shared knowledge, resources and expertise. Within this framework, long-term, structured teacher–student relationships must be developed.

The present study was limited by the fact that it considered a course within only one programme of study. However, the field experiment and the specifics of the educational programme determined this choice. An additional limitation is that no questions tested students’ knowledge of the institutional context and energy crisis. Furthermore, the study sample was small (i.e., 66 students), and some of the results may have been influenced by student non-attendance. Nonetheless, one of the strengths of the study is that the model can be easily replicated in similar settings and in other disciplines, perhaps while modifying/adding certain questionnaire items to suit the relevant programme of study.

Complex challenges require clear strategies with synergy between senior and junior parties. Students are at the heart of the university, and young people are at the heart of future civil society.

## Supplementary Information

Supplementary Information 1.

Supplementary Information 2.

## Data Availability

​All data generated or analysed during this study are included in this published article [and its supplementary information files].
