# Supplementary material for: Sustainable education and youth confidence as pillars of future civil society
Source: Sci Rep. 2023 Jan 18;13:955. doi: 10.1038/s41598-023-28143-9 (PMC9849327; doi:10.1038/s41598-023-28143-9)
Supplement: Supplementary file 2 — Supplementary Information 2. [file 41598_2023_28143_MOESM2_ESM.docx]

**GENERAL PART. DATA IN PERCENTAGE.**

| **1** | **Indicate your A.Y. in which you started your master's degree** | END | START |
| --- | --- | --- | --- |
|  | 2020-2021 | 21 | 20 |
|  | 2021-2022 | 45 | 43 |
|  | Other [More] | 0 | 1 |
| **2** | **How old are you** | END | START |
|  | 22 | 16 | 16 |
|  | 23 | 16 | 15 |
|  | 24 | 11 | 13 |
|  | 25 | 18 | 16 |
|  | More | 5 | 4 |
| **3** | **Indicate your gender** | END | START |
|  | Man | 42 | 40 |
|  | Woman | 24 | 24 |
|  | I prefer not to answer | 0 | 0 |
| **4** | **In which area of Italy did you study in high school?** | END | START |
|  | Northern Italy | 1 | 1 |
|  | Central Italy | 56 | 53 |
|  | Southern Italy | 9 | 10 |
|  | Other [More] | 0 | 0 |
| **5** | **Indicate your occupation** | END | START |
|  | Student | 40 | 42 |
|  | Student and worker | 12 | 11 |
|  | Student and part-time worker (seasonal) | 14 | 11 |
| **6** | **Indicate [It indicates] who you live with** | END | START |
|  | With your family | 54 | 50 |
|  | With roommates | 11 | 13 |
|  | With your partner(s) | 1 | 0 |
|  | On my own | 0 | 1 |
| **7** | **How do you define your character?** | END | START |
|  | Selfish | 0 | 1 |
|  | More selfish than altruistic | 4 | 5 |
|  | Neither selfish nor altruistic | 14 | 9 |
|  | More altruistic than selfish | 40 | 42 |
|  | Altruistic | 8 | 7 |
| **8** | **Are you thinking more about the present or the future?** | END | START |
|  | Present | 12 | 17 |
|  | Future | 41 | 35 |
|  | Undecided | 13 | 12 |

**BEHAVIORAL PART. DATA IN PERCENTAGE.**

| **9** | **What does sustainability concern?** | END | START |
| --- | --- | --- | --- |
|  | Environmental aspects | 0 | 6 |
|  | Economic aspects | 0 | 0 |
|  | Social aspects | 0 | 0 |
|  | A mix of social, economic and environmental aspects | 66 | 58 |
| **10** | **How often do you spend time as a volunteer [much time you devote to volunteering]?** | END | START |
|  | Never | 26 | 32 |
|  | Rarely | 22 | 16 |
|  | Sometimes | 17 | 13 |
|  | Often | 0 | 3 |
|  | Always | 1 | 0 |
| **11** | **How often [many times] do you make recycling collection?** | END | START |
|  | Never | 0 | 0 |
|  | Rarely | 1 | 4 |
|  | Sometimes | 4 | 6 |
|  | Often | 21 | 19 |
|  | Always | 40 | 35 |
| **12** | **How often do you [do] practice sport? [exercises]** | END | START |
|  | Never | 1 | 1 |
|  | Rarely | 8 | 7 |
|  | Sometimes | 17 | 21 |
|  | Often | 20 | 19 |
|  | Always | 20 | 16 |
| **13** | **How often do you buy sustainable products?** | END | START |
|  | Never | 2 | 1 |
|  | Rarely | 9 | 19 |
|  | Sometimes | 37 | 28 |
|  | Often | 18 | 16 |
|  | Always | 0 | 0 |
| **14** | **How often do you opt for [make] green choices in [with] transportation?** | END | START |
|  | Never | 9 | 7 |
|  | Rarely | 16 | 22 |
|  | Sometimes | 19 | 12 |
|  | Often | 19 | 20 |
|  | Always | 3 | 3 |
| **15** | **How often do you need to be in touch with nature?** | END | START |
|  | Never | 0 | 0 |
|  | Rarely | 5 | 9 |
|  | Sometimes | 19 | 19 |
|  | Often | 35 | 30 |
|  | Always | 7 | 6 |
| **16** | **How many hours do you walk on average in a day, excluding weekends?** | END | START |
|  | less than one km | 7 | 6 |
|  | between one and three kilometers | 29 | 37 |
|  | between three and six kilometers | 22 | 16 |
|  | more than six kilometers | 8 | 5 |

**SPECIFIC (SUSTAINABLE AND ENERGY) PART. DATA IN PERCENTAGE.**

| **17** | **How much would you pay for a kWh produced from fossil sources (cent€/kWh)?** | END | START |
| --- | --- | --- | --- |
|  | 5 | 13 | 8 |
|  | 10 | 20 | 14 |
|  | 15 | 14 | 20 |
|  | 20 | 12 | 16 |
|  | 25 | 6 | 4 |
|  | 30 | 0 | 0 |
|  | 35 | 1 | 2 |
| **18** | **How much would you pay for a kWh produced from renewable sources (cent€/kWh)?** | END | START |
|  | 5 | 2 | 2 |
|  | 10 | 6 | 9 |
|  | 15 | 18 | 14 |
|  | 20 | 13 | 16 |
|  | 25 | 16 | 15 |
|  | 30 | 10 | 6 |
|  | 35 | 1 | 2 |
| **19** | **How much would you sell a kWh produced from fossil sources (cent€/kWh)?** | END | START |
|  | 5 | 10 | 7 |
|  | 10 | 15 | 19 |
|  | 15 | 14 | 13 |
|  | 20 | 11 | 14 |
|  | 25 | 6 | 4 |
|  | 30 | 6 | 3 |
|  | 35 | 4 | 4 |
| **20** | **How much would you sell a kWh produced from renewable sources (cent€/kWh)?** | END | START |
|  | 5 | 4 | 1 |
|  | 10 | 12 | 15 |
|  | 15 | 12 | 16 |
|  | 20 | 14 | 18 |
|  | 25 | 13 | 7 |
|  | 30 | 10 | 5 |
|  | 35 | 1 | 2 |
| **21** | **Energy communities are important. Do you share this view?** | END | START |
|  | strongly disagree | 0 | 0 |
|  | disagree | 0 | 3 |
|  | undecided | 2 | 8 |
|  | agree | 33 | 40 |
|  | strongly agree | 31 | 13 |
| **22** | **Sustainable certifications are important. Do you share this view?** | END | START |
|  | strongly disagree | 0 | 1 |
|  | disagree | 1 | 5 |
|  | undecided | 2 | 8 |
|  | agree | 38 | 34 |
|  | strongly agree | 25 | 16 |
| **23** | **Subsidies for the production of green sources are important. Do you share this view?** | END | START |
|  | strongly disagree | 0 | 0 |
|  | disagree | 0 | 2 |
|  | undecided | 1 | 3 |
|  | agree | 33 | 28 |
|  | strongly agree | 32 | 31 |
| **24** | **Fossil fuel subsidies are important. Do you share this view?** | END | START |
|  | strongly disagree | 19 | 8 |
|  | disagree | 24 | 20 |
|  | undecided | 13 | 24 |
|  | agree | 8 | 8 |
|  | strongly agree | 2 | 4 |
| **25** | **What value do you recognize to the subsidy for green energy produced and self-consumed (cent€/kWh)?** | END | START |
|  | 0 | 0 | 0 |
|  | 1 | 0 | 1 |
|  | 2 | 2 | 0 |
|  | 3 | 9 | 10 |
|  | 4 | 15 | 19 |
|  | 5 | 25 | 22 |
|  | 6 | 15 | 12 |
| **26** | **What value do you recognize to the subsidy for fossil energy produced and self-consumed (cent€/kWh)?** | END | START |
|  | 0 | 14 | 9 |
|  | 1 | 17 | 13 |
|  | 2 | 12 | 13 |
|  | 3 | 19 | 15 |
|  | 4 | 2 | 9 |
|  | 5 | 2 | 2 |
|  | 6 | 0 | 3 |
| **27** | **Green sources reduce geopolitical risks. Do you share this view?** | END | START |
|  | strongly disagree | 1 | 1 |
|  | disagree | 2 | 6 |
|  | undecided | 17 | 22 |
|  | agree | 31 | 24 |
|  | strongly agree | 15 | 11 |
| **28** | **Green sources produce competitive advantage for enterprises. Do you share this view?** | END | START |
|  | strongly disagree | 0 | 0 |
|  | disagree | 1 | 4 |
|  | undecided | 8 | 16 |
|  | agree | 37 | 36 |
|  | strongly agree | 20 | 8 |
| **29** | **Are you in favor of penalties/taxes for companies that do not follow sustainability principles?** | END | START |
|  | strongly disagree | 0 | 0 |
|  | disagree | 3 | 1 |
|  | undecided | 6 | 9 |
|  | agree | 28 | 28 |
|  | strongly agree | 29 | 26 |
| **30** | **Are you in favor of penalties/taxes for citizens who do not follow principles of sustainability?** | END | START |
|  | strongly disagree | 0 | 2 |
|  | disagree | 10 | 4 |
|  | undecided | 6 | 15 |
|  | agree | 33 | 33 |
|  | strongly agree | 17 | 10 |
| **31** | **Sustainable education is a pillar of civil society. Do you share this view?** | END | START |
|  | strongly disagree | 0 | 0 |
|  | disagree | 0 | 0 |
|  | undecided | 0 | 4 |
|  | agree | 23 | 24 |
|  | strongly agree | 43 | 36 |
| **32** | **Green sources reduce environmental impact. Do you share this opinion?** | END | START |
|  | strongly disagree | 0 | 0 |
|  | disagree | 0 | 2 |
|  | undecided | 1 | 9 |
|  | agree | 35 | 29 |
|  | strongly agree | 30 | 24 |
| **33** | **The development of new professionals is necessary for the ecological transition. Do you share this view?** | END | START |
|  | strongly disagree | 0 | 0 |
|  | disagree | 0 | 1 |
|  | undecided | 3 | 8 |
|  | agree | 29 | 32 |
|  | strongly agree | 34 | 23 |
| **34** | **I use green sources and no longer fossil sources, so I modify my consumption habits to take advantage of the potential economic benefits. Do you share this view?** | END | START |
|  | strongly disagree | 0 | 0 |
|  | disagree | 3 | 2 |
|  | undecided | 12 | 18 |
|  | agree | 37 | 33 |
|  | strongly agree | 14 | 11 |
| **35** | **I use green sources and no longer fossil sources, so I can also consume more because the unit environmental impact is reduced. Do you share this view?** | END | START |
|  | strongly disagree | 16 | 7 |
|  | disagree | 28 | 27 |
|  | undecided | 11 | 15 |
|  | agree | 10 | 11 |
|  | strongly agree | 1 | 4 |
| **36** | **The younger generation (students in university) are able to implement a sustainable development plan in practice. Do you share this opinion?** | END | START |
|  | strongly disagree | 0 | 1 |
|  | disagree | 9 | 6 |
|  | undecided | 15 | 13 |
|  | agree | 35 | 33 |
|  | strongly agree | 7 | 11 |
| **37** | **New generations (students in high school) are able to implement a sustainable development plan in practice. Do you share this opinion?** | END | START |
|  | strongly disagree | 8 | 5 |
|  | disagree | 14 | 13 |
|  | undecided | 21 | 20 |
|  | agree | 22 | 25 |
|  | strongly agree | 1 | 1 |
| **38** | **Internet use affects sustainability. Do you share this view?** | END | START |
|  | strongly disagree | 0 | 0 |
|  | disagree | 3 | 3 |
|  | undecided | 9 | 13 |
|  | agree | 44 | 36 |
|  | strongly agree | 10 | 12 |
| **39** | **Greenwashing helps sustainable development. Do you share this opinion?** | END | START |
|  | strongly disagree | 19 | 14 |
|  | disagree | 18 | 7 |
|  | undecided | 13 | 25 |
|  | agree | 15 | 16 |
|  | strongly agree | 1 | 2 |
| **40** | **I am willing to self-produce green energy at my home even without receiving incentives, that is, neither explicitly (through incentives) nor implicitly (through exemptions, tax deductions, etc.) How much do you agree with this statement ?** | END | START |
|  | strongly disagree | 1 | 1 |
|  | disagree | 9 | 7 |
|  | undecided | 18 | 26 |
|  | agree | 33 | 25 |
|  | strongly agree | 5 | 5 |
| **41** | **For the purpose of achieving climate neutrality, how important do you think it is to use methodologies that increase energy efficiency with respect to the development of energy production from renewable sources ?** | END | START |
|  | More important | 7 | 0 |
|  | Equally important | 43 | 44 |
|  | Less important | 12 | 8 |
|  | Impossible to say a priori | 4 | 8 |
| **42** | **Do you think the decarbonization of a system like Italy's is achievable through the development and application of new technologies?** | END | START |
|  | Yes | 5 | 2 |
|  | Yes, as long as all possible uses are electrified | 2 | 6 |
|  | Yes, as long as technologies currently under development are also made competitive, in addition to the massive use of electricity | 28 | 27 |
|  | No, a change in our behaviors is also necessary | 28 | 22 |
|  | I don't know. | 3 | 7 |
| **43** | **Which one, among these stakeholder categories, does most influence sustainable development?** | END | START |
|  | workers | 0 | 2 |
|  | consumers | 12 | 9 |
|  | general society | 28 | 24 |
|  | local community | 1 | 2 |
|  | enterprises (value chain actors) | 25 | 27 |
